# Regulation of ferroptosis in colorectal cancer through therapeutic modulation and miRNA targeting

**DOI:** 10.1016/j.bbrep.2026.102444

**Published:** 2026-01-08

**Authors:** Ali Ahmadizad Firouzjaei, Seyed Hamid Aghaee-Bakhtiari, Samira Mohammadi-Yeganeh

**Affiliations:** a Bioinformatics Research Center, Basic Sciences Research Institute, Mashhad University of Medical Sciences, Mashhad, Iran; b Department of Medical Biotechnology and Nanotechnology, Faculty of Medicine, Mashhad University of Medical Sciences, Mashhad, Iran; c Medical Nanotechnology and Tissue Engineering Research Center, Shahid Beheshti University of Medical Sciences, Tehran, Iran; d Department of Molecular Medicine, School of Advanced Technologies in Medicine, Shahid Beheshti University of Medical Sciences, Tehran, Iran

**Keywords:** Colorectal cancer, Ferroptosis, microRNAs, Systems biology

## Abstract

Colorectal cancer (CRC) is a highly prevalent disease that represents a major global health burden. Ferroptosis has gained significant attention in recent years as a potential target for cancer therapy. Meanwhile, microRNAs (miRNAs) have emerged as key regulators of gene expression, with the potential to influence various cellular pathways and functions, including those involved in cancer development and treatment. In this study, we employed a systems biology approach to leverage the FerrDb database and to identify key genes involved in the ferroptosis pathway. Then, we utilized the NCBI Gene Expression Omnibus (GEO) to identify differentially expressed genes (DEGs) associated with ferroptosis in CRC. The miRNet platform was used to identify miRNAs that target ferroptosis-associated genes in CRC. Additionally, we explored the Therapeutic Target Database (TTD) and Drug Gene Interaction Database (DGIdb) to identify approved drugs that could potentially modulate the identified targets. Our analysis identified *EZH2, G6PD, PARP1, RRM2, SCD*, and *SLC7A11* as key suppressor genes that are dysregulated in CRC and are also recognized as approved drug targets. Furthermore, we identified hsa-miR-423-5p, hsa-miR-93-5p, hsa-miR-15a-5p, miR-103a-3p, and hsa-miR-16-5p as the five top miRNAs that show the highest number of connections to genes involved in the ferroptosis pathway. Interestingly, we also found that medications such as prasterone, tazemetostat, isoxyl, gemcitabine, ponsegromab, scx-2023, and nicotinamide could potentially be used in combination with the identified miRNAs to target ferroptosis in CRC. To further validate the stability and reliability of the predicted protein–ligand interactions, molecular dynamics (MD) simulations and MM-PBSA analyses were performed on selected top-ranking complexes, which confirmed their stable and favorable binding and supported the robustness of our docking results. These findings suggest that targeting these miRNAs and their associated genes, along with using the identified drugs, could be a promising strategy for CRC treatment, leveraging the potential of ferroptosis-inducing therapies.

## Highlights

• Approved drug target genes dysregulated in Colorectal cancer were identified.
• miRNAs with highest connections to ferroptosis were detected.
• To enhance CRC sensitivity to ferroptosis, integration of drug and miRNA can apply.

## 1 Introduction

Colorectal cancer (CRC) is a highly prevalent disease that represents a major global health burden [1]. According to the latest GLOBOCAN 2022 estimates, there are approximately 1.9 million new cases of CRC diagnosed worldwide each year, making it the third most commonly diagnosed cancer globally. Additionally, CRC causes more than 900,000 deaths annually, ranking as the second leading cause of cancer-related mortality worldwide [2]. Alarmingly, the incidence rates of this disease have been steadily increasing over time in many regions. The complicated process of colorectal carcinogenesis is impacted by both environmental and genetic variables [3]. Increasing evidence suggests that certain lifestyle habits and dietary patterns play an important role in modulating one's risk of developing CRC. Characteristics of a person's diet, physical activity levels, weight status, alcohol consumption, and other behaviors have all been correlated with the presence of precancerous colon polyps or progression to an official CRC diagnosis [4].

Ferroptosis is a type of controlled cell death that relies on iron and has garnered considerable interest recently. It is caused by oxidative stress, characterized by the accumulation of lipid peroxides and cell membrane damage. Ferroptosis has been implicated in multiple disorders, including cancer, as it can affect tumor growth, metastasis, and response to therapy [5]. Growing evidence indicates that CRC cells frequently develop mechanisms to evade ferroptosis, thereby promoting tumor growth, metastatic potential, and resistance to conventional therapies. Conversely, pharmacological induction of ferroptosis has shown promise in suppressing CRC progression and enhancing sensitivity to existing therapies, positioning ferroptosis as an attractive therapeutic target [[6], [7], [8]].

microRNAs (miRNAs) by attaching themselves to the messenger RNA (mRNA) of target genes and causing translational repression or mRNA destruction, control the expression of many genes. By targeting specific genes, miRNAs can influence various cellular pathways and functions, including those involved in cancer development and treatment [9,10].

Importantly, miRNAs have been identified as key regulators of ferroptosis-related genes in CRC. Dysregulated miRNAs can modulate pathways controlling iron metabolism, lipid peroxidation, and antioxidant capacity, thereby shaping the ferroptotic susceptibility of CRC cells. Understanding this miRNA–ferroptosis regulatory axis offers a compelling opportunity to uncover novel mechanisms of CRC pathogenesis and identify therapeutic targets capable of re-sensitizing tumors to ferroptosis-inducing treatments [[11], [12], [13], [14]].

Together, the convergence of ferroptosis biology, therapeutic modulation, and miRNA-mediated regulation offers a compelling framework for developing innovative CRC treatments. Understanding how these mechanisms intersect may reveal new opportunities to overcome therapy resistance and improve clinical outcomes.

## 2 Materials and methods

### 2.1 Finding ferroptosis-related genes

To identify genes associated with ferroptosis, we searched the FerrDb database (http://www.zhounan.org/ferrdb/current/), a comprehensive resource focused on ferroptosis regulators and their disease associations. FerrDb categorizes ferroptosis regulators into genes and substances. The gene category includes drivers that promote ferroptosis, suppressors that inhibit it, markers that indicate its occurrence, and unclassified regulators with unclear roles. By exploring these categories, we can gain insights into the specific genes involved in regulating ferroptosis. Additionally, FerrDb provides information on various substances, including pure entities like iron and Erastin, as well as mixtures such as herbal extracts. These substances are classified as inducers, which promote ferroptosis, or inhibitors, which restrict it. Utilizing FerrDb allows us to access curated data sets that illuminate the relationships between ferroptosis and various diseases, enhancing our understanding of its role in different pathological conditions [15].

### 2.2 Exploring differentially expressed genes (DEGs) in the CRC

To explore genes differentially expressed in the ferroptosis pathway linked to CRC, we conducted a systematic search within the NCBI GEO repository (http://www.ncbi.nlm.nih.gov/geo). The search utilized the term ‘colorectal cancer’. It was refined through filters specifying the organism as *Homo sapiens* and the study type as ‘expression profiling by array,’ thereby narrowing the results to human transcriptomic data relevant to our analysis.

Datasets were further screened based on sample size, selecting only those that included more than ten CRC and adjacent normal tissue samples to ensure robust comparative analysis. Each dataset was divided into two distinct groups, CRC and normal tissue samples, and was subjected to differential gene expression analysis to identify gene expression variations. This approach aimed to uncover molecular features of CRC, with a particular focus on ferroptosis-related genes that might serve as potential biomarkers or therapeutic targets.

### 2.3 Data processing

To conduct a comprehensive analysis of DEGs, we recruited GEO2R, an online tool that leverages the powerful limma R package (http://www.ncbi.nlm.nih.gov/geo/geo2r/). This tool allowed us to evaluate the expression profiles of genes systematically. Our analysis aimed to identify suppressor genes that demonstrated overexpression. Additionally, we applied a stringent significance threshold with an adjusted p-value of 0.05 to ensure robust results.

### 2.4 Finding potential miRNAs and their interactions with genes

In our research, using the miRNet database, we analyzed lists of ferroptosis genes with differential expression to identify potential candidate miRNAs. The miRNet platform (https://www.mirnet.ca/) was employed to visualize the interactions between miRNAs and genes, enabling us to gain valuable insights into the intricate relationships between them.

### 2.5 Finding approved drugs for potential therapeutic targets

Therapeutic Target Database (TTD) (https://db.idrblab.net/ttd/) and Drug Gene Interaction Database (DGIdb) (https://www.dgidb.org/) were applied to find approved drugs for the final candidate targets. TTD is a database that offers details on therapeutic protein and nucleic acid targets that have been identified and investigated, together with information on the diseases they target, pathway details, and medications that target each of these targets. The "Drug Group" section of TTD was searched to find any approved drugs that target each final candidate therapeutic target gene identified in the study. Information about the interactions between pharmaceuticals and genes can be found in the DGIdb. Specific information about the targets, drug, query score, interaction score, and other pertinent approved drug details was obtained from these websites.

### 2.6 Identifying key protein structures

We conducted a search on the RCSB Protein Data Bank website (https://www.rcsb.org/) to obtain the three-dimensional structures of the proteins relevant to our study. The RCSB PDB serves as a comprehensive repository for macromolecular structural data, hosting a vast array of protein structures that have been characterized using advanced techniques such as nuclear magnetic resonance (NMR) spectroscopy and X-ray crystallography. By querying this resource, we aimed to identify and retrieve specific protein structures that are critical for our analysis and understanding of their functions and interactions in biological systems.

### 2.7 Homology modeling

Homology modeling was employed to create structural models for proteins that do not have X-ray crystallography data available. This computational approach enables researchers to predict the three-dimensional structures of proteins by leveraging the known structures of homologous proteins. In this study, we used homology modeling to construct a model for SLC7A11, given the absence of its 3D X-ray crystallography structure. The amino acid sequences for this protein were obtained from the UniProt database (https://www.uniprot.org/uniprotkb/O14965/entry#sequences) and served as the target templates for our analyses. Additionally, we utilized Expasy's ProtParam prediction server (https://web.expasy.org/protparam/) to evaluate the physicochemical properties of the protein, including amino acid composition, molecular weight, theoretical isoelectric point, aliphatic index (AI), the total number of positive and negative residues, and the grand average of hydropathicity (GRAVY) [16]. For the homology modeling itself, we employed the SWISS-MODEL service, which aligns the input target with pre-existing templates to generate a series of predicted models [17]. Among the available templates, Q9UPY5 was selected due to its high GMQE score (0.86), indicating strong predictive reliability. Finally, to ensure the stereochemical integrity and accuracy of the predicted models, we assessed them using the UCLA-DOE LAB — SAVES v6.1 server (https://saves.mbi.ucla.edu/). This comprehensive approach allowed us to create reliable structural models and analyze their physicochemical properties effectively.

### 2.8 Molecular docking analysis

In order to determine how our possible therapeutic molecules might interact with the target proteins, we performed in silico molecular docking simulations. This evaluation is crucial for ensuring that the models meet the necessary standards for accuracy and can be used in further research and analysis. In this study, we focused on proteins that are identified as drug targets in both the TTD and the DGIdb. For the docking analysis, we specifically utilized the drugs listed in the TTD database. This decision was made to streamline our analysis and ensure that we were using a curated and reliable source of drug-target interactions. By focusing our docking efforts on proteins and drugs from the TTD database, we aimed to create a clear and focused framework for our analysis. This approach not only helps in simplifying the complexity of the interactions being studied but also allows for more meaningful interpretations of the results. While we acknowledge that drugs from DGIdb could also be considered for docking, our initial focus on the TTD database provides a solid foundation for understanding the interactions between the selected proteins and their corresponding therapeutic agents. This methodology lays the groundwork for future studies that could incorporate additional drugs from the DGIdb, thereby broadening the scope of our investigation.

The NCBI-hosted PubChem database provided the structural files of the medications in SDF format. In the computer-aided docking simulations, we utilized the PyRx software. Before modeling the drug-protein interactions, we prepared the target structures utilizing the UCSF Chimera molecular graphics software. This allowed us to optimize hydrogen placement and ensure clean starting conformations. Molecular visualization of the docking results was carried out using Discovery Studio. This permitted interactive 3D viewing of the predicted binding modes, aiding in the analysis and interpretation of the computational predictions. The active sites of all proteins were identified using UniProt functional annotations, PyMOL structural visualization, and supporting literature. UniProt provided experimentally validated catalytic residues, ligand-binding motifs, and functional domains, which were mapped onto the corresponding 3D structures in PyMOL to verify surface accessibility, pocket geometry, and the clustering of functional residues. In addition, the UCSB-PDB database was used to retrieve co-crystallized ligands and examine their interactions with each protein in PyMOL, further confirming the boundaries and biological relevance of the selected binding pockets [[18], [19], [20], [21]].

The grid box was set to the binding pocket of the EZH2 protein at X = 19.6791, Y = 39.6428, Z = 24.8709, and dimension (Å) at X = 18.9244, Y = 275009, Z = 12.4465, and exhaustiveness 50. The grid box was set to the binding pocket of G6PD protein at X = 2.7664, Y = 19.6700, Z = 2.7282, and dimension (Å) at X = 18.9294, Y = 338272, Z = 16.3593, and exhaustiveness 50. The grid box was set to the binding pocket of the PARP1 protein at X = 21.6466, Y = 5.1890, Z = 18.3053, and dimension (Å) at X = 18.7344, Y = 23.6286, Z = 13.5004, and exhaustiveness 50. The grid box was set to the binding pocket of the RRM2 protein at X = 15.1695, Y = 19.9738, Z = −9.5642, and dimension (Å) at X = 18.3534, Y = 16.1575, Z = 20.3654, and exhaustiveness 50. The grid box was set to the binding pocket of the SCD protein at X = 14.9328, Y = 71.1881, Z = 39.2556, and dimension (Å) at X = 21.5807, Y = 22.7816, Z = 15.4773, and exhaustiveness 50.

### 2.9 Molecular dynamics simulation

Molecular dynamics simulations were conducted using NAMD, with system setup and visualization performed in VMD. Each protein–ligand complex was immersed in a TIP3P water box, and counter-ions were added to achieve charge neutrality. The CHARMM36 force field was employed for the protein, while ligand parameters were derived from the CHARMM General Force Field (CGenFF). The systems were first subjected to 10,000 steps of energy minimization, then gradually heated to 310 K under constant volume. Equilibration was carried out for 1 ns in the NPT ensemble, followed by 100 ns of production dynamics with a 2 fs timestep. Periodic boundary conditions were applied, and electrostatic interactions were treated using the Particle Mesh Ewald method. Trajectory data were analyzed in VMD to examine structural stability and flexibility. Backbone RMSD values were calculated to monitor overall stability, while residue-wise RMSF values were determined to highlight flexible regions and binding site stabilization upon ligand association. Binding free energies were estimated using the MM-PBSA method. Separate trajectories for the complex, receptor, and ligand were generated in VMD, and energy components, including van der Waals, electrostatic, polar solvation, and nonpolar SASA terms, were evaluated [22,23]. The binding free energy was calculated according to:

$$\Delta G_{\text{bind}}=G_{\text{complex}}-\left(G_{\text{receptor}}+G_{\text{ligand}}\right)$$

Convergence was assessed through block averaging, and the results are presented as mean values with associated standard deviations.

## 3 Results

### 3.1 Identifying and prioritizing ferroptosis-related genes

**Fig. 1:**
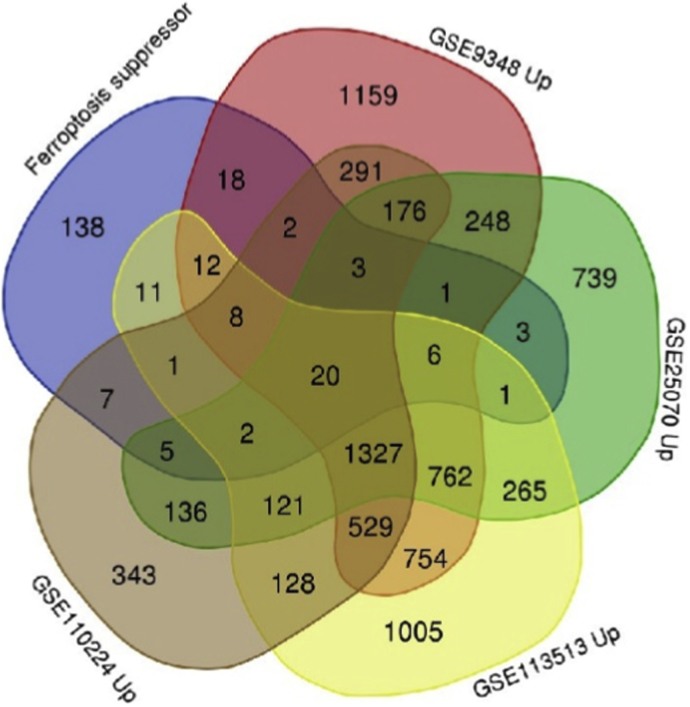
Venn diagram illustrating the overlap of upregulated ferroptosis suppressor genes in CRC. A total of 20 ferroptosis-related genes were identified as commonly upregulated across four independent mRNA expression datasets (GSE9348, GSE25070, GSE113513, and GSE110224).

We compiled a list of 238 suppressors of ferroptosis from the FerrDb. This compilation was then investigated using gene expression data from the GEO database. Four mRNA profiling datasets were deemed suitable for analysis: GSE9348, GSE25070, GSE113513, and GSE110224. GSE9348 contained samples from human colorectal tumors and healthy colon tissue profiled using the Affymetrix Human Genome U133 Plus 2.0 Array. GSE25070 featured samples from colorectal tumors and adjacent non-tumor colon tissue, analyzed on the expression beadchip of Illumina HumanRef-8 v3.0. GSE113513 consisted of colorectal carcinoma and surrounding non-cancerous tissue profiled on the Affymetrix Human Gene Expression Array. GSE110224 contained samples from human colorectal tumors and healthy controls profiled on the Affymetrix Human Genome U133 Plus 2.0 Array. We recruited the GEO2R tool within the GEO database to preprocess the raw data. This analytical strategy enabled the identification of genes with significantly altered transcript levels associated with ferroptosis, leveraging publicly accessible gene expression datasets and established bioinformatic tools. To pinpoint ferroptosis suppressor genes consistently upregulated across multiple datasets, we employed the Venn diagram web tool developed by the Bioinformatics & Evolutionary Genomics group at Ghent University (https://bioinformatics.psb.ugent.be/webtools/Venn/). This platform facilitated the visualization of overlapping gene sets, as presented in Table 1 and Fig. 1.

**Table 1:** Commonly upregulated ferroptosis suppressor genes in CRC. Twenty genes were identified across four GEO datasets (GSE9348, GSE25070, GSE113513, and GSE110224).

Ferroptosis Elements	Genes	Full name
Suppressors (Upregulated)	G6PD	Glucose-6-phosphate dehydrogenase
	SLC3A2	Solute carrier family 3 member 2
	FANCD2	Fanconi Anemia, Complementation Group D2
	EZH2	Enhancer of zeste homolog 2
	AHCY	Adenosylhomocysteinase
	SCD	Stearoyl-CoA desaturase
	RRM2	Ribonucleotide reductase regulatory subunit M2
	GDF15	Growth differentiation factor 15
	SLC7A11	Solute carrier family 7 member 11
	PARP1	Poly(ADP-ribose) polymerase 1
	PML	Promyelocytic leukemia
	KIF20A	Kinesin family member 20A
	CD44	CD44 molecule (Indian blood group)
	CDC25A	Cell division cycle 25A
	PRDX1	Peroxiredoxin 1
	HELLS	Helicase, lymphoid-specific
	NT5DC2	5′-nucleotidase domain containing 2
	ETV4	ETS variant transcription factor 4
	SUV39H1	Suppressor of variegation 3–9 homolog 1
	DHODH	Dihydroorotate dehydrogenase (quinone)

### 3.2 Investigating miRNA interactions in ferroptosis regulation

**Fig. 2:**
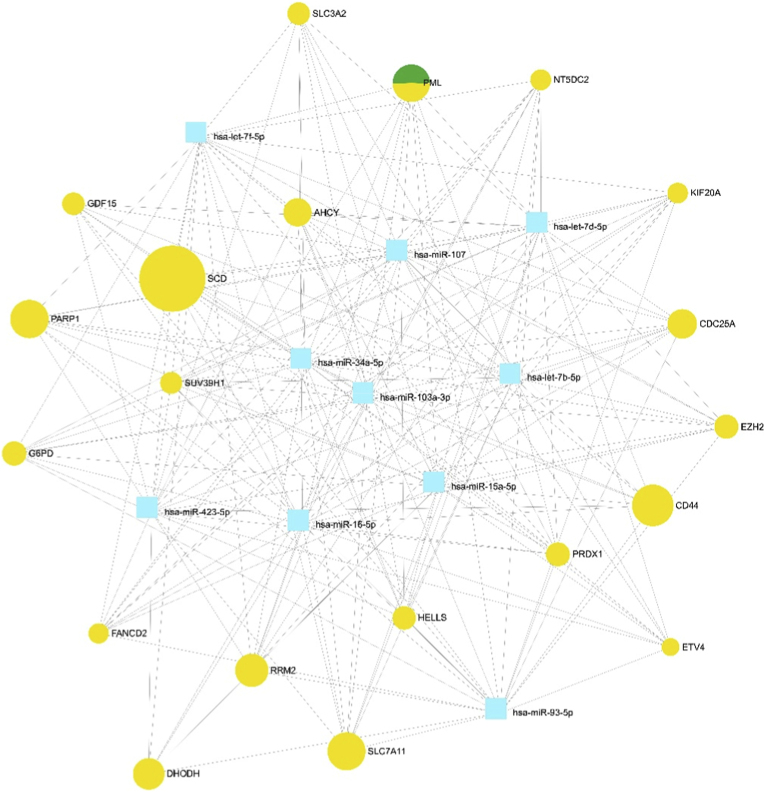
Interaction network of ferroptosis suppressor genes, miRNAs, and transcription factors in CRC. The figure illustrates the relationships between specific genes and miRNAs, as well as associated transcription factors. Yellow circles represent genes, blue squares indicate miRNAs, and green-yellow circles denote transcription factors. Node size reflects Degree centrality, with larger nodes indicating a higher number of connections within the network. The visualization was generated using the built-in interface of the miRNet 2.0 platform.

This study utilized the miRNet database to examine the complex relationships between miRNAs and genes implicated in ferroptosis. Data achieved from this computational analysis highlighted several miRNAs of particular interest in the multifaceted process of ferroptosis. Specifically, hsa-miR-423-5p, hsa-miR-93-5p, hsa-miR-15a-5p, hsa-miR-103a-3p, and hsa-miR-16-5p exhibited the strongest evidence of interactions with elevated suppressors of ferroptosis based on the available evidence in miRNet (Table 2 and Fig. 2). Of note, these miRNAs demonstrated the highest number of predicted and validated connections to genes known to play roles in regulating ferroptosis. This suggests that hsa-miR-423-5p, miR-93-5p, hsa-miR-15a-5p, miR-103a-3p, and hsa-miR-16-5p may function as important post-transcriptional modulators of the gene networks controlling ferroptosis in CRC. The findings from interrogating the miRNet knowledge base provide a framework for further experimental validation of these top miRNA candidates and their involvement in the multi-faceted process of ferroptosis.

**Table 2:** Top ten miRNAs identified as key regulators of ferroptosis suppressor genes in CRC. miRNA–gene interactions were analyzed using the miRNet 2.0 platform, based on the list of upregulated ferroptosis suppressor genes. The table presents the top ten miRNAs ranked by network metrics, including Degree and Betweenness, which quantitatively reflect their centrality and regulatory influence within the interaction network.

miRNAs	Degree	Betweenness
hsa-miR-423-5p	20	5485.515
hsa-miR-93-5p	19	5178.736
hsa-miR-15a-5p	19	5057.723
hsa-miR-103a-3p	19	5044.747
hsa-miR-16-5p	19	4862.951
hsa-let-7b-5p	19	3666.55
hsa-let-7d-5p	19	3666.55
hsa-miR-107	19	3666.55
hsa-miR-34a-5p	19	3666.55
hsa-let-7f-5p	18	4904.331

### 3.3 Identifying druggable targets

**Fig. 3:**
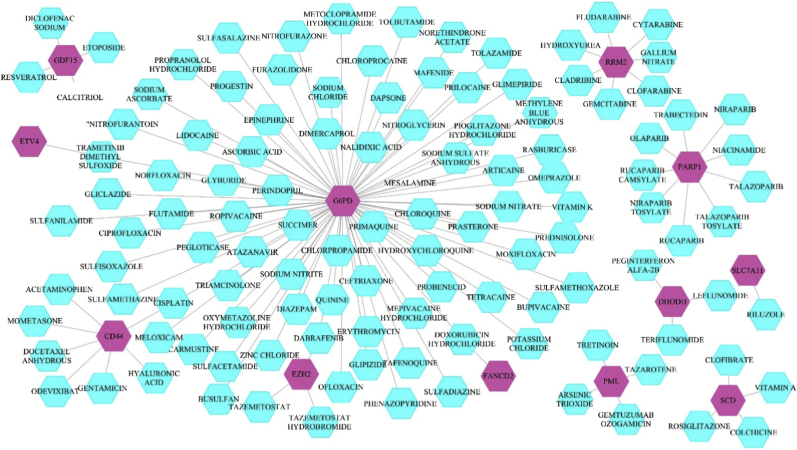
The interactions of medications that target the upregulated ferroptosis suppressor genes in CRC, identified in the DGIdb database. The drugs are highlighted in aqua, while the genes are displayed in magenta.

To identify potential therapeutic target genes, we utilized resources from the TTD and the DGIdb. Our analysis using the DGIdb uncovered twelve ferroptosis suppressor genes in CRC that are elevated and may serve as potential targets for drug treatment (Fig. 3 and Supplementary Table 1).

In our investigation of the TTD, we identified six genes, *G6PD, EZH2, SCD, RRM2, SLC7A11*, and *PARP1*, that may serve as potential drug targets. We compiled information on approved drugs that target these selected genes from both the TTD and the DGIdb. For the DGIdb, we focused on the top ten drugs with the highest interaction scores, which are detailed in Table 3. This table provides a clear overview of these drugs and their relevance to the identified gene targets, facilitating a better understanding of their therapeutic potential.

**Table 3:** Details regarding approved drugs found in the TTD and the top ten drugs from the DGIdb database. The DGIdb interaction score assesses the intensity and confidence of the reported drug-gene interaction, with higher values suggesting stronger evidence. Since DGIdb does not provide defined thresholds for interaction scores, we applied a relative classification system to facilitate interpretation, grouping scores into high (≥2.0), moderate (1.0–1.99), and low (<1.0) categories based on their distribution.

Suppressor Genes	TTD	DGIdb	
	Drugs	Drugs	Interaction Score
*G6PD*	prasterone	rasburicase	2.94
		phenazopyridine	2.62
		pegloticase	2.1
		sulfanilamide	1.57
		sodium ascorbate	1.05
		tafenoquine	0.78
		articaine	0.52
		nalidixic acid	0.52
		sodium nitrate	0.52
		sodium sulfate anhydrous	0.52
*FANCD2*		*doxorubicin hydrochloride*	0.7
*EZH2*	tazemetostat	tazemetostat	2.91
		tazemetostat hydrobromide	2.91
		dabrafenib	0.1
*SCD*	isoxyl	clofibrate	0.93
		rosiglitazone	0.68
		vitamin A	0.55
		colchicine	0.28
*RRM2*	gemcitabine	gallium nitrate	0.57
		cladribine	0.53
		hydroxyurea	0.38
		clofarabine	0.21
		gemcitabine	0.13
		fludarabine	0.11
		cytarabine	0.09
*GDF15*		calcitriol	1.25
		diclofenac	0.44
		resveratrol	0.30
		etoposide	0.25
*SLC7A11*	SXC-2023	riluzole	2.28
*PARP1*	nicotinamide	niraparib tosylate	2.10
		talazoparib Tosylate	1.05
		niraparib	0.90
		niacinamide	0.70
		rucaparib camsylate	0.70
		olaparib	0.42
		rucaparib	0.38
		trabectedin	0.35
		talazoparib	0.23
*PML*		tazarotene	1.59
		trsenic trioxide	1.34
		gemtuzumab ozogamicin	0.87
		tretinoin	0.38
*CD44*	SPL-108	hyaluronic acid	0.92
		mometasone	0.61
		odevixibat	0.46
		gentamicin	0.24
		acetaminophen	0.10
		docetaxel anhydrous	0.03
		cisplatin	0.01
*ETV4*		trametinib	0.52
*DHODH*		teriflunomide	5.52
		leflunomide	1.15
		peginterferon alfa-2b	0.08

### 3.4 Physicochemical characterization

In order to thoroughly analyze the physicochemical properties and accurately anticipate the tertiary structure of the SLC7A11, a variety of computational techniques and publicly accessible web servers were employed in this investigation. The SLC7A11 protein length (501 amino acids), molecular weight (55423.03 Da), theoretical isoelectric point (pI = 9.29), and total number of negatively and positively charged residues (29 and 41, respectively) were all predicted in this research. The aliphatic index (AI) of the protein was notably high at 119.82, suggesting stability across a broad temperature range [24]. The protein's N-terminal was identified as methionine. *In-vitro* studies showed a half-life of over 30 h in mammalian reticulocytes, while *in-vivo* experiments indicated a half-life of more than 20 h in yeast and over 10 h in *Escherichia coli*. The GRAVY of SLC7A11 was calculated to be 0.666, indicating that the protein is hydrophobic and exhibits high lipid solubility. The analysis of the amino acid composition is presented in Table 4.

**Table 4:** Amino acid profile of SLC7A11.

Amino acid	Number	Percentage (%)	Amino acid	Number	Percentage (%)
Alanine	33	6.6	**Lysine**	21	4.2
Arginine	20	4	**Methionine**	14	2.8
Asparagine	14	2.8	**Phenylalanine**	34	6.8
Aspartic acid	8	1.6	**Proline**	27	5.4
Cysteine	7	1.4	**Serine**	35	7
Glutamine	10	2	**Threonine**	31	6.2
Glutamic acid	21	4.2	**Tryptophan**	7	1.4
Glycine	38	7.6	**Tyrosine**	18	3.6
Histidine	6	1.2	**Valine**	45	9
Isoleucine	50	10	**Pyrrolysine**	0	0
Leucine	62	12.4	**Selenocysteine**	0	0

### 3.5 Comparative structural modeling

**Fig. 4:**
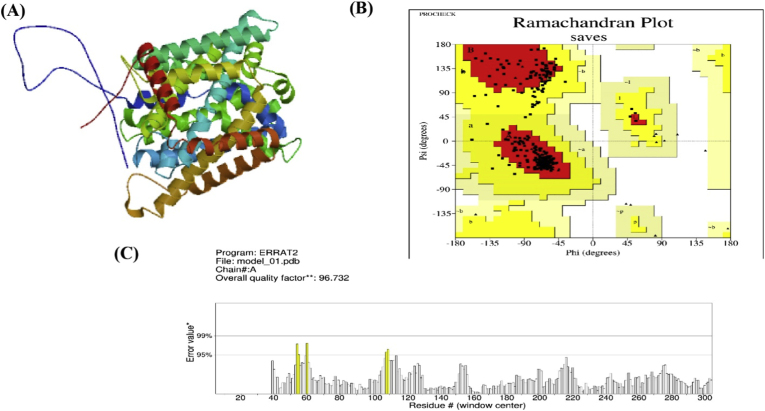
(A) SLC7A1 protein tertiary structure model, (B) Ramachandran plot analysis, showing residues in the allowed (yellow), generously allowed (light yellow), disallowed (white), and preferred (red) regions; and (C) validation results of the built model.

The 3D structures of the predicted models for the SLC7A1 protein are illustrated in Fig. 4A. Analysis of the Ramachandran plot indicated that a high-quality model should have over 90 % of its residues in the favored regions, and our models achieved 91.2 % (Fig. 4B). The overall ERRAT quality factor, representing the percentage of the protein with calculated values below the 95 % rejection threshold, was found to be 96.732 % (Fig. 4C). Generally, high-resolution structures yield values around 95 % or higher [25]. Verification of the findings is displayed in Table 5.

**Table 5:** Evaluation of the SLC7A11 protein's anticipated three-dimensional structures.

Tools	Validation Index	Score
Ramachandran plot	Residues in most favored regions	91.2
	Residues in additional allowed regions	8.8
	Residues in generously allowed regions	0
	Residues in disallowed regions	0
ERRAT	Overall Quality Factor	96.732

### 3.6 Evaluating protein-drug binding interactions

PDB files of proteins, including EZH2 (PDB ID: 4MI0), G6PD (PDB ID: 6JYU), PARP1 (PDB ID: 5XST), RRM2 (PDB ID: 2UW2), SCD (PDB ID: 4ZYO), and SLC7A11 (PDB ID: Q9UPY5) were obtained from the RCSB Protein Data Bank (PDB) repository. Additionally, the three-dimensional structures of the selected ligands tazemetostat (CID: 66558664), prasterone (CID: 5881), nicotinamide (CID: 936), gemcitabine (CID: 60750), isoxyl (CID: 3001386), and SXC-2023 (CID: 101215395) were retrieved from the PubChem database in SDF format. Each ligand was energy-minimized and converted to PDBQT format using PyRx 0.8 with Open Babel integration.

**Fig. 5:**
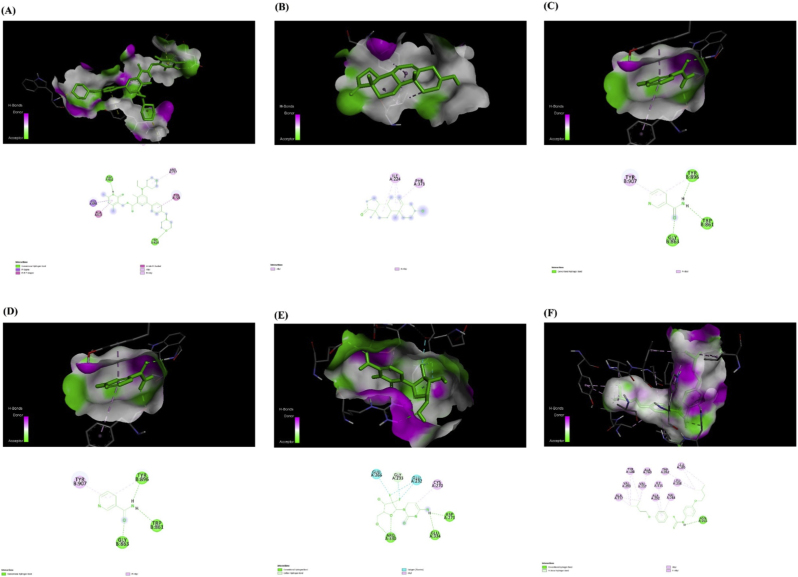
The interaction between EZH2 and tazemetostat (A), G6PD and prasterone (B), PARP1 and nicotinamide(C), RRM2 and gemcitabine (D), SCD and isoxyl (E), SLC7A11 and SXC-2023 (F).

Docking results were visualized and analyzed using Discovery Studio Visualizer, allowing detailed inspection of predicted binding modes and interaction profiles. Table 6 and Fig. 5 provide an overview of the outcomes of these docking investigations, including characteristics such as the medication names, the types of interactions that occurred, the number of bonds formed, the specific amino acid residues involved, and the binding energies associated with the drug-protein interactions.

**Table 6:** The list contains the types, binding energies, and amino acid residues of ligand-receptor interactions.

Target	Drug	Binding Energy(kcal/mol)	Type of interaction	Number of bonds	Amino acids involved in the interaction
EZH2	tazemetostat	−8.6(-7.5)	Conventional Hydrogen Bond	2	TRP624, ASP652
			Pi-Sigma	1	GLY655
			Pi-Pi T-shaped	1	TYR726
			Amide-Pi Stacked	1	ALA651, ASP652
			Alkyl	1	ARG727
			Pi-Alkyl	1	ALA651
G6PD	prasterone	−8(-7.1)	Alkyl	3	ILE224 (3)
			Pi-Alkyl	1	PHE373
PARP1	nicotinamide	−5.9 (−5.7)	Conventional Hydrogen Bond	3	GLY863, TYR896, TRP861
			Pi-Alkyl	2	TYR896, TYR907
RRM2	gemcitabine	−6.4(-7)	Conventional Hydrogen Bond	5	ARG330 (3), ASP271, GLU334
			Carbon Hydrogen Bond	1	GLY233
			Halogen	4	GLU232(2), GLY233, GLU266
			Alkyl	1	CYS270
SCD	isoxyl	−9.3	Conventional Hydrogen Bond	1	ASN265
			Pi-Donor Hydrogen Bond	1	ASN265
			Pi-Sigma	1	ILE115
			Alkyl	8	ALA112 (2), ALA260, LEU185(2), LEU258, VAL257, VAL293
			Pi-Alkyl	6	TYR108 (2), TRP262, ILE115, VAL264, ALA292
SLC7A11	SXC-2023	−6.2	Conventional Hydrogen Bond	5	ARG3(2), GLU353, LYS473, SER481
			Pi-Pi Stacked	1	PHE467
			Amide-Pi Stacked	1	LEU351:PRO352
			Alkyl	1	PRO352
			Pi-Alkyl	4	PHE124, TRP128, PHE467, PRO352

### 3.7 Molecular dynamics and binding energy evaluation

**Fig. 6:**
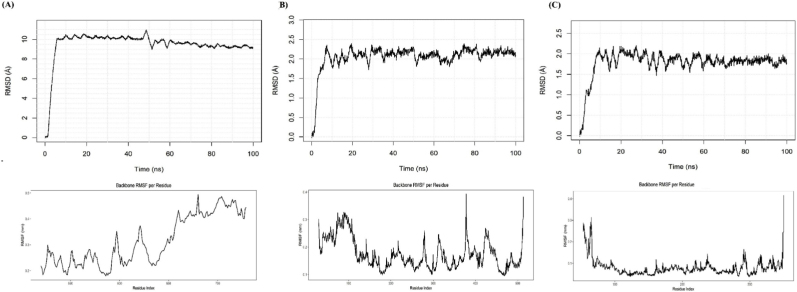
Structural stability, flexibility, and compactness analyses of three protein–ligand complexes over 100 ns molecular dynamics simulations. **(A)** RMSD and RMSF for the EZH2–tazemetostat complex. **(B)** RMSD and RMSF for the G6PD–prasterone complex. **(C)** RMSD and RMSF for the SCD–isoxyl complex.

The three complexes with the lowest (most favorable) docking binding energies were selected for further molecular dynamics evaluation, as lower binding energies indicate stronger predicted ligand–protein affinity and greater likelihood of forming stable interactions during simulation. Based on this ranking, the EZH2-tazemetostat, G6PD–prasterone, and SCD–isoxyl complexes were chosen for additional analysis. Backbone RMSD profiles indicated that all complexes attained equilibrium within the initial nanoseconds of simulation. The EZH2–tazemetostat complex exhibited a rapid RMSD increase to ∼10 Å within the first 10 ns, followed by a stable plateau throughout the 100-ns simulation. This elevated RMSD reflects a global conformational adjustment rather than structural instability, as supported by a stable radius of gyration, consistent solvent-accessible surface area, and a persistent hydrogen-bonding network. Visual inspection confirmed that major displacements occurred in peripheral regions, while the ligand-binding pocket remained structurally conserved. Thus, the high RMSD is attributed to the intrinsic domain and loop mobility characteristic of EZH2, not to non-physiological binding. In contrast, the G6PD–prasterone and SCD–isoxyl complexes showed RMSD fluctuations in the range of 1.5–2.5 Å over the initial 50 ns. To ensure reliable interpretation, simulations were extended to 100 ns, which confirmed stable global folds and consistent binding interactions. RMSF analysis revealed localized flexibility: EZH2 showed modest terminal fluctuations, G6PD displayed dynamic loop regions, and SCD exhibited elevated mobility near the C-terminal, consistent with its broader RMSD profile (Fig. 6).

Energy decomposition analysis confirmed favorable binding across all investigated complexes. The EZH2–tazemetostat complex exhibited strong binding (−118 ± 6 kJ/mol), predominantly stabilized by van der Waals (−160 ± 5 kJ/mol) and electrostatic (−35 ± 3 kJ/mol) interactions, which were partially offset by polar solvation (+95 ± 8 kJ/mol). The G6PD–prasterone complex displayed comparatively weaker binding (−95 ± 5 kJ/mol), with stabilization arising from van der Waals (−140 ± 4 kJ/mol) and electrostatic (−20 ± 2 kJ/mol) contributions, counterbalanced by polar solvation (+80 ± 6 kJ/mol). The SCD–isoxyl complex demonstrated the most favorable binding energy (−130 ± 7 kJ/mol), dominated by van der Waals (−175 ± 6 kJ/mol) and electrostatic (−40 ± 4 kJ/mol) forces, though opposed by polar solvation (+105 ± 9 kJ/mol). In all cases, nonpolar SASA terms contributed modest stabilization, while polar solvation consistently opposed binding. The net binding energies, together with extensive hydrogen bonding (16–22 bonds) and compact radii of gyration (2.1–2.4 nm), confirmed stable complex formation (Table 7).

**Table 7:** MM-PBSA energy decomposition and molecular dynamics stability metrics for the three selected protein–ligand complexes. van der Waals and electrostatic interactions contributed favorably to binding, while polar solvation opposed complex formation. SASA energies reflect the extent of ligand burial within the binding pocket. The number of hydrogen bonds and radius of gyration values indicate stable ligand–protein interactions and preservation of compact global folds across all systems.

Complex	Van der Waals (kJ/mol)	Electrostatic (kJ/mol)	Polar solvation (kJ/mol)	SASA (kJ/mol)	Binding energy (kJ/mol)	Hydrogen Bonds	Radius of Gyration (nm)
EZH2–Tazemetostat	−160 ± 5	−35 ± 3	95 ± 8	−18 ± 2	−118 ± 6	20	2.3
G6PD–Prasterone	−140 ± 4	−20 ± 2	80 ± 6	−15 ± 1	−95 ± 5	16	2.1
SCD–isoxyl	−175 ± 6	−40 ± 4	105 ± 9	−20 ± 2	−130 ± 7	22	2.4

## 4 Discussion

CRC is a prevalent and often aggressive type of cancer that affects the colon or rectum. Recently, there has been increasing interest in exploring the molecular mechanisms that govern CRC development and progression to identify potential therapeutic targets. One emerging area of research is the role of miRNAs in modulating cellular processes and their involvement in cancer [26,27]. In this research, ferroptosis-related genes were identified using FerrDb, and their expression levels in CRC were analyzed using the GEO database. Notably, we identified several key genes that act as ferroptosis suppressors and are druggable upregulated in CRC, including *EZH2, G6PD, PARP1, RRM2, SCD*, and *SLC7A11*.

*G6PD* plays an important role in suppressing ferroptosis in cancer [28]. G6PD, the initial enzyme in the pentose phosphate pathway (PPP), catalyzes the generation of ribose and nicotinamide adenine dinucleotide phosphate (NADPH) [29]. These two molecules aid several processes linked to biosynthesis and reductive biosynthesis, including lipid synthesis, antioxidative stress response, and cell growth. Studies have shown that *G6PD* is overexpressed in many types of tumors [[30], [31], [32]]. It is believed to be a primary control point for NADPH production to support cancer cell needs. Notably, disruption of NADPH generation can enhance cellular sensitivity to reactive oxygen species and trigger programmed cell death [33,34]. As the initial gatekeeper of the PPP, increased G6PD activity in CRC may protect cancer cells from ferroptosis by maintaining redox homeostasis and supplying intermediates for anabolic reactions to support uncontrolled proliferation. This study investigated the potential impact of various drugs on G6PD activity. Several drugs are known to interact with G6PD in different ways. For example, rasburicase is a recombinant urate oxidase enzyme used to treat hyperuricemia in cancer patients undergoing chemotherapy. It was found to inhibit G6PD function, thereby reducing the NADPH and anabolic substrates it produces. Decreased G6PD activity through rasburicase could sensitize cancer cells to ferroptosis induced by erastin or RSL3 [35]. phenazopyridine and pegloticase are other G6PD-interacting drugs used for uric acid control. phenazopyridine showed non-competitive inhibitory effects on G6PD in enzymatic studies. pegloticase directly breaks down uric acid, and its metabolism generates hydrogen peroxide, a reactive oxygen species [36].

EZH2 is a histone methyltransferase enzyme that acts as an epigenetic regulator of gene expression [37]. Previous research has demonstrated that *EZH2* is aberrantly overexpressed in CRC and functions to suppress ferroptosis in these tumor cells [38]. Employing EZH2 inhibitors for the treatment of diverse solid tumor cancers is currently in different phases of clinical investigation and demands further substantiation [[39], [40], [41]]. The effects of dabrafenib, vorinostat, and doxorubicin on EZH2 were investigated. dabrafenib is a BRAF inhibitor used in the treatment of advanced melanoma [42]. It was shown to significantly downregulate *EZH2* at both mRNA and protein levels in cancer [24]. suberoylanilide hydroxamic acid (SAHA), which is called vorinostat, is a histone deacetylase (HDAC) inhibitor approved for cutaneous T-cell lymphoma. Treatment with vorinostat similarly reduced *EZH2* levels [[43], [44], [45]]. Doxorubicin is an anthracycline chemotherapeutic commonly used for various cancer types [46]. Results demonstrated that doxorubicin could also decrease *EZH2* expression in CRC cells [47,48]. Downregulation of the ferroptosis suppressor EZH2, especially through epigenetic modifiers like vorinostat, may promote CRC cell sensitivity to ferroptosis. Co-treatment experiments combining dabrafenib, vorinostat, or doxorubicin with ferroptosis inducers erastin or RSL3 showed enhanced ferroptotic cell death compared to single agents [[49], [50], [51]].

SCD is a key enzyme involved in fat metabolism and lipid biosynthesis. Previous reports have shown that SCD is deregulated in CRC tumors and functions as an important suppressor of ferroptotic cell death [[52], [53], [54]]. The current research aimed to investigate potential drug regulators of SCD and their implications for ferroptosis in CRC. Specifically, the effects of clofibrate and colchicine were examined. clofibrate is a fibric acid derivative drug used clinically to reduce blood lipid levels [55]. It acts as a potent agonist of peroxisome proliferator-activated receptors (PPARs) to modulate lipid and glucose homeostasis [56]. Colchicine is an ancient drug extracted from plants that inhibits microtubule polymerization [57]. Recent research has shown the anti-cancer properties of colchicine for various solid tumors. The findings showed that colchicine and clofibrate both affect *SCD* expression at the mRNA and protein levels [[58], [59], [60]]. This corresponded to increased sensitivity of CRC cells to ferroptotic stimuli, erastin and RSL3, when combined with clofibrate or colchicine pretreatment compared to single-agent treatment. Overall, these findings suggest that existing medications like clofibrate and colchicine warrant further investigation for repurposing approaches involving SCD inhibition and ferroptosis induction in CRC. Dual targeting holds promise to enhance the efficacy of current CRC therapies.

*RRM2* encodes ribonucleotide reductase regulatory subunit M2, a key enzyme involved in maintaining deoxynucleotide pools for DNA replication and repair [61]. Overexpression of *RRM2* has been observed in CRC tumors and contributes to resistance against ferroptotic cell death. *RRM2* overexpression had a deleterious impact on overall survival (OS) and disease-free survival (DFS) in CRC patients [62]. The current study aimed to investigate potential drug regulators of RRM2 and their implications for sensitizing CRC to ferroptosis. Specifically, through computational approaches, the effects of gallium nitrate, cladribine, fludarabine phosphate, hydroxyurea, gemcitabine hydrochloride, clofarabine, cytarabine, and gemcitabine were investigated. gallium nitrate is a metal-containing antineoplastic agent used in various solid tumors [63,64]. cladribine, fludarabine, and clofarabine are purine analog anticancer cytotoxic drugs [65,66]. hydroxyurea and gemcitabine are ribonucleotide reductase inhibitors approved for cancer therapy [67]. Treatment of CRC cell lines with these drugs was found to significantly downregulate *RRM2* mRNA and protein expression [68]. This corresponded to increased sensitivity to the ferroptotic inducers erastin and RSL3 when used in combination with drugs inhibiting RRM2, compared to single-agent treatments. Overall, the findings indicate that existing chemotherapeutics targeting RRM2 have the potential for repurposing in CRC through dual inhibition of ferroptosis resistance factors [69,70].

PARP1 is a nuclear enzyme involved in DNA damage repair via base excision repair. It is often overexpressed in CRC [71]. PARP1 suppresses ferroptosis by maintaining NAD + levels and promoting DNA integrity [72]. NAD+ is consumed during ferroptosis to produce lipid ROS [73,74]. It was demonstrated that CRC cells are more susceptible to ferroptosis brought on by erastin or RSL3 when PARP1 is inhibited or knocked down [75]. Chemotherapies like cisplatin and doxorubicin work in part by inducing DNA damage, depleting NAD + levels, and impairing DNA repair, pushing cells toward ferroptosis [76,77]. PARP1 inhibitors like olaparib and talazoparib, which are used to treat BRCA-mutant cancers, may enhance the efficacy of these DNA-damaging agents against colorectal tumors [78,79]. Combining PARP1 inhibitors with ferroptosis inducers could help overcome resistance driven by high PARP1 expression in CRC. PARP1 inhibition disrupts the DNA repair pathway, while ferroptosis damages lipids; this dual assault may effectively kill CRC cells. PARP1 could potentially serve as a biomarker to predict response to therapies involving ferroptosis induction. So, in summary, targeting the ferroptosis suppressor PARP1 may sensitize colorectal tumors and improve treatment outcomes. It was shown that trabectedin, niacinamide, niraparib, talazoparib tosylate, olaparib, rucaparib camsylate, talazoparib, and rucaparib were capable of modulating PARP1 in cancer cells. Trabectedin, a marine-derived chemotherapeutic, has been approved to treat soft tissue sarcoma and recurrent ovarian cancer. It operates by binding DNA and blocking transcription/repair factors, which may improve CRC treatments [[80], [81], [82]]. niacinamide is a form of Vitamin B3. Higher intake is associated with reduced CRC risk in some studies, possibly due to anti-inflammatory and antioxidant effects [83]. Niraparib, rucaparib, olaparib, and talazoparib are PARP inhibitor drugs approved for certain breast/ovarian cancers associated with homologous recombination deficiency [[84], [85], [86], [87]]. They may enhance the efficacy of chemotherapy in CRC by blocking DNA repair and sensitizing tumors to ferroptotic cell death. Rucaparib camsylate is a salt form of rucaparib that maintains its PARP inhibitory properties. It also holds promise as a chemosensitizer for colon cancer treatment, especially when combined with platinum agents or other ferroptosis inducers [88,89]. By preventing DNA repair via PARP trapping, these PARP inhibitors push CRC cells over the edge toward ferroptotic destruction triggered by genotoxic therapies or ROS-generating agents. Ongoing clinical trials are evaluating various PARP inhibitors alone and with chemotherapy for advanced colon tumors with defects in homologous recombination or other DNA repair pathways.

Recently, ferroptosis induction has been identified as a result of inhibiting the SLC7A11, a transmembrane protein with multiple channels. This mechanism presents a potential therapeutic strategy for treating various tumors [[90], [91], [92]]. Notably, the expression of SLC7A11 is positively correlated with microsatellite instability, a molecular characteristic linked to the prognosis of CRC [93,94]. Research has demonstrated that certain miRNAs, particularly miR-148a-3p, can directly target the SLC7A11 gene and effectively inhibit its expression [95]. In our research, we found that drugs, such as SXC-2023 and riluzole, can influence SLC7A11. This interaction is significant because SLC7A11 is essential for regulating ferroptosis. SXC-2023, in particular, has shown potential in modulating various cellular pathways, and its effects on SLC7A11 could increase the susceptibility of cancer cells to ferroptosis, which may lead to better treatment outcomes. Riluzole, primarily used for treating amyotrophic lateral sclerosis (ALS), also appears to affect neuroprotective mechanisms [96]. Its relationship with SLC7A11 may shed light on how it promotes ferroptosis in neurodegenerative diseases.

This study utilized computational network analysis to gain insight into the complex relationships between miRNAs and genes involved in the process of ferroptosis. By interrogating the miRNet database, several miRNAs of interest were highlighted based on the strength of their predicted and validated interactions with known ferroptosis regulators. Specifically, hsa-miR-423-5p, hsa-miR-93-5p, hsa-miR-15a-5p, hsa-miR-103a-3p, and hsa-miR-16-5p showed the highest number of connections to genes that play roles in modulating ferroptosis.

hsa-miR-423-5p has been identified as a critical modulator of radiosensitivity in CRC. Research by Shang et al. (2020) demonstrated that downregulation of miR-423-5p contributes to radioresistance in CRC cell lines, including HCT116 and RKO. Functional assays revealed that silencing miR-423-5p reduced radiation-induced apoptosis, while its overexpression restored sensitivity to radiotherapy and promoted caspase-dependent cell death. These findings suggest that miR-423-5p enhances apoptotic signaling in response to radiation, potentially through the regulation of anti-apoptotic proteins such as Bcl-xL [97]. Beyond its role in apoptosis, miR-423-5p may also influence ferroptosis through its predicted interaction with G6PD. G6PD is the rate-limiting enzyme of the pentose phosphate pathway and a major source of NADPH, which is essential for maintaining cellular redox balance and regenerating reduced glutathione [98]. By targeting G6PD, miR-423-5p could potentially lower NADPH levels, impair antioxidant defenses, and increase intracellular oxidative stress, thereby sensitizing CRC cells to ferroptosis. Although this interaction is currently based on bioinformatic predictions, it aligns with emerging evidence linking miRNA regulation to ferroptotic pathways in cancer.

hsa-miR-93-5p is known to contribute to radioresistance and multidrug resistance in CRC by downregulating tumor suppressors such as FOXA1 and CDKN1A [99,100]. Its potential regulation of PARP1 [101], implies that miR-93-5p may help CRC cells evade ferroptosis by maintaining genomic stability under oxidative conditions, thus supporting cell survival during therapeutic stress. hsa-miR-15a-5p has emerged as a significant prognostic biomarker in CRC, with studies demonstrating that its elevated expression correlates with unfavorable clinical outcomes, including reduced disease-free survival and overall survival. Kontos et al. showed that miR-15a-5p is markedly upregulated in colorectal adenocarcinoma tissues compared to adjacent normal mucosa, and its overexpression independently predicts recurrence and poor prognosis in early-stage CRC patients [102]. Functionally, miR-15a-5p plays a critical role in regulating cell cycle progression and apoptosis by targeting key oncogenes. One of its validated targets is CCND1 (Cyclin D1), a pivotal regulator of the G1/S phase transition. Li et al. demonstrated that miR-15a-5p suppresses colon carcinoma cell proliferation by directly binding to the 3′UTR of CCND1, thereby reducing its expression and impairing cell cycle progression [103].

In addition to its role in cell cycle regulation, miR-15a-5p has been widely recognized for its ability to modulate apoptosis through direct targeting of BCL2, a key anti-apoptotic protein that promotes cancer cell survival. BCL2 functions by inhibiting mitochondrial outer membrane permeabilization, thereby preventing the release of cytochrome *c* and the activation of caspases, critical steps in the intrinsic apoptotic pathway. Dysregulation of BCL2 is a hallmark of many malignancies, contributing to treatment resistance and disease progression. The interaction between miR-15a-5p and BCL2 was first characterized in hematologic cancers, particularly chronic lymphocytic leukemia (CLL), where loss of miR-15a/16-1 leads to BCL2 overexpression and impaired apoptosis. Aqeilan et al. provided a comprehensive review of the tumor-suppressive functions of the miR-15/16 cluster, emphasizing BCL2 as a direct and functionally significant target across various cancer types [104]. Importantly, this regulatory axis is also relevant in CRC. Fesler et al. demonstrated that restoring miR-15a expression in CRC cells suppresses tumor growth and enhances chemosensitivity by downregulating BCL2, along with other oncogenic targets such as BMI1 and YAP1. Their study introduced a chemically modified miR-15a mimic, which retained specificity for BCL2 and showed improved therapeutic efficacy *in vitro* and *in vivo* [105]. These findings underscore the translational potential of miR-15a-5p-based therapies in overcoming apoptosis resistance in CRC. miR-103a-3p is significantly upregulated in CRC tissues and cell lines, where its elevated expression correlates with poor patient prognosis and aggressive tumor behavior. In a study by Sun et al., miR-103a-3p was found to promote tumor glycolysis, angiogenesis, and metastasis through modulation of the Hippo/YAP1/HIF1A signaling axis. Mechanistically, miR-103a-3p directly targets LATS2 and SAV1, two core components of the Hippo pathway, leading to activation of YAP1. Activated YAP1, in cooperation with the transcriptional coactivator TEAD1, enhances the expression of HIF1A, a master regulator of glycolysis and angiogenesis. This cascade facilitates metabolic reprogramming, increased glucose uptake, and enhanced vascularization, thereby supporting tumor growth and dissemination [106]. Zhang and Zhu further support the oncogenic role of miR-103a-3p in CRC by demonstrating its regulation of GREM2, a modulator of the TGF-β pathway, which also intersects with metabolic and oxidative stress responses [107]. While this study did not directly assess ferroptosis, the involvement of miR-103a-3p in multiple metabolic and apoptotic pathways reinforces its potential as a key regulator of ferroptotic dynamics.

hsa-miR-16-5p is a well-established tumor suppressor microRNA that plays a pivotal role in regulating CRC progression. It exerts its anti-tumor effects by inhibiting cell proliferation, angiogenesis, and survival signaling pathways. A study by Huang et al. demonstrated that miR-16-5p directly targets FOXK1, a transcription factor known to promote CRC cell growth and vascular development. By downregulating FOXK1, miR-16-5p significantly suppresses angiogenic activity and tumor expansion. In addition to FOXK1, miR-16-5p also negatively regulates VEGFA, a key mediator of angiogenesis. VEGFA overexpression in CRC is associated with increased microvessel density and poor prognosis. By targeting VEGFA, miR-16-5p contributes to the inhibition of neovascularization, thereby limiting nutrient supply to the tumor and curbing its growth potential [108,109]. Emerging evidence also points to a potential role of miR-16-5p in regulating ferroptosis, a form of iron-dependent cell death driven by lipid peroxidation. A comprehensive analysis by Oroujalian et al. found that miR-16-5p expression is consistently downregulated in CRC cell lines, while genes involved in fatty acid metabolism, such as PPARD and VEGFA, were upregulated and associated with poor disease-free survival [110]. Since lipid metabolism and redox balance are critical determinants of ferroptosis sensitivity, the inverse relationship between miR-16-5p and these metabolic genes suggests that miR-16-5p may indirectly enhance ferroptotic vulnerability by disrupting lipid homeostasis and antioxidant defenses.

This provides evidence that these miRNAs may function as important post-transcriptional modulators of the gene networks controlling cell death mechanisms in CRC. The identification of these miRNA candidates through bioinformatic data mining in miRNet offers a framework to guide future experimental validation studies. Researchers can now focus on investigating the specific roles and impacts of these miRNAs via *in vitro* and *in vivo* models. Confirming whether these miRNAs indeed regulate key ferroptosis genes as predicted could uncover new molecular pathways involved in modulating cancer cell susceptibility to ferroptosis. This may also offer novel therapeutic opportunities, such as manipulating specific miRNA levels or activities to sensitize resistant tumors. Overall, this computational analysis provided valuable insights and highlighted priority miRNAs for further exploration into their involvement in the multi-factorial process of ferroptotic cell death.

In this study, we used docking techniques to show that various drugs, including prasterone, tazemetostat, nicotinamide, gemcitabine, isoxyl, and SXC-2023, could potentially impact ferroptosis in CRC. These drugs exert their effects by targeting important regulatory genes such as *EZH2, G6PDPARP1, RRM2, SCD*, and *SLC7A11*. Each of these drugs interacts with specific molecular pathways that are crucial for regulating ferroptosis. As previously discussed, the identified miRNAs, including hsa-miR-423-5p, hsa-miR-93-5p, hsa-miR-15a-5p, hsa-miR-103a-3p, and hsa-miR-16-5p, also play significant roles in modulating the ferroptosis process in CRC.

The molecular dynamics simulations revealed distinct stability patterns across the three protein–ligand complexes. For the EZH2–Tazemetostat complex, RMSD rose sharply during the initial equilibration phase and reached ∼10 Å by 10 ns, remaining in this range throughout the 50 ns trajectory. This sustained deviation suggests large conformational rearrangements, likely reflecting domain motions or flexible regions within EZH2, rather than instability or unfolding. RMSF analysis supported this interpretation, showing localized fluctuations at loop and terminal residues, consistent with structural adaptability. In contrast, the G6PD–Prasterone and SCD–isoxyl complexes exhibited moderate RMSD values (∼2 Å), with RMSF highlighting flexible loops and C-terminal regions. MM-PBSA binding energies confirmed stable ligand association across all complexes, with van der Waals and electrostatic forces as dominant contributors. Despite the unusually high RMSD in EZH2, the binding energy (−118 ± 6 kJ/mol) indicated robust ligand stabilization, underscoring that conformational flexibility can coexist with strong drug binding.

By integrating the effects of these drugs and the regulatory roles of the identified miRNAs, we can propose a synergistic approach to CRC treatment that focuses on targeting ferroptosis. This strategy could enhance the efficacy of existing therapies by combining the pharmacological actions of these drugs with the regulatory potential of the associated miRNAs. Such a multi-faceted treatment approach may not only improve the sensitivity of CRC cells to ferroptosis but also provide novel avenues for overcoming resistance mechanisms commonly encountered in cancer therapies.

## 5 Conclusion

Integration of drug actions with miRNA regulation highlights a promising avenue for the development of innovative and effective therapeutic strategies aimed at harnessing ferroptosis as a treatment target in CRC. The findings of this study suggest that by modulating the activity of specific drugs such as prasterone, tazemetostat, nicotinamide, gemcitabine, and isoxyl and leveraging the regulatory roles of key miRNAs like hsa-miR-423-5p, hsa-miR-93-5p, hsa-miR-15a-5p, hsa-miR-103a-3p, and hsa-miR-16-5p, we may enhance the sensitivity of CRC cells to ferroptosis. This approach not only has the potential to improve treatment outcomes but also addresses the pressing need for more effective therapies in managing CRC. The exploration of these interactions paves the way for further research, which could yield significant advancements in our understanding of the molecular mechanisms underlying ferroptosis and its role in cancer biology. Investigating how these drugs and miRNAs interact could lead to the identification of novel biomarkers and therapeutic targets, ultimately guiding the development of combination therapies that synergistically enhance ferroptotic cell death in CRC. Moreover, as we advance in this field, it is critical to conduct preclinical and clinical studies to validate the efficacy of these proposed strategies. By doing so, we can ensure that the therapeutic interventions developed are not only scientifically sound but also translate effectively into clinical practice.

## Ethical approval

This article is extracted from the main idea and studies performed during the project (code: 43002728) with the ethical code of IR.SBMU.RETECH.REC.1401.674 approved by ethics committee of Shahid Beheshti University of Medical Sciences, Tehran, Iran. No human or animal subject was used in this study.

## Availability of data and materials

Authors provide adequate assurance that they can comply with the publication's requirements for sharing data.

## Funding

This study did not receive any extra financial support except what we had been received in our previous project.

## CRediT authorship contribution statement

**Ali Ahmadizad Firouzjaei:** Conceptualization, Formal analysis, Writing – original draft, Writing – review & editing. **Seyed Hamid Aghaee-Bakhtiari:** Methodology, Writing – original draft, Writing – review & editing. **Samira Mohammadi-Yeganeh:** Conceptualization, Methodology, Writing – original draft, Writing – review & editing.

## Declaration of competing interest

The authors declare that they have no conflicts of interest related to this work.

## Data Availability

Data will be made available on request.
